# Effects of Micro-Osteoperforation Depths on Canine Retraction Rate and Root Resorption: A Systematic Review and Meta-Analysis

**DOI:** 10.1055/s-0045-1806932

**Published:** 2025-05-02

**Authors:** Potjanakorn Inpanya, Pannapat Chanmanee, Supontep Teerakanok

**Affiliations:** 1 Orthodontic Section, Department of Preventive Dentistry, Faculty of Dentistry, Prince of Songkla University, Songkhla, Thailand; 2 Periodontic Section, Department of Conservative Dentistry, Faculty of Dentistry, Prince of Songkla University, Songkhla, Thailand

**Keywords:** orthodontics, micro-osteoperforation, tooth movement, meta-analysis, systematic review

## Abstract

This systematic review and meta-analysis aimed to compare canine retraction rates and the amounts of root resorption in different depths of micro-osteoperforations (MOPs) during canine retraction in orthodontic patients. Relevant literature was sought using a prespecified search strategy until May 2024. Electronic medical and scientific databases included PubMed/MEDLINE, Scopus, EMBASE, Web of Science, and the Cochrane's Library (clinical trials). The review protocol was registered in Prospero (CRD42024555722). The data were analyzed in terms of mean difference for comparison using a random-effect meta-analysis. A total of 14 randomized controlled trial studies were included. According to the findings of the meta-analysis on MOPs and their impact on the mean rate of canine movement, the MOP groups showed a significantly higher rate compared with the control groups (weighted mean difference = 0.32; 95% confidence interval [CI], 0.24–0.40; *p* = 0.00 and weighted mean difference = 0.20; 95% CI, 0.01–0.40; *p* = 0.04) at depths of 2 to 4 and 5 to 7 mm, respectively. Three studies reported no differences in root resorption between the MOP groups and the control groups. Both MOP depths, that is, 2 to 4 and 5 to 7 mm, accelerated canine retraction more than the controls by approximately 0.32 and 0.20 mm/month, respectively. However, both MOP depths presented root resorption during canine retraction that was not different from the controls.

## Introduction

Over time, various surgical techniques have been developed to accelerate orthodontic tooth movement, improving treatment efficiency and reducing overall treatment duration. Several cortical bone penetration techniques are designed to cut through the cortical bone and reach the cancellous bone, leading to transient osteopenia, which results in a reduction in bone density and decreased resistance to tooth movement. 1 Frost defined this condition as the regional acceleratory phenomenon (RAP) in 1983. 2

In 2001, Wilcko et al reported two case reports involving patients with severe crowding malocclusion. These patients underwent orthodontic treatment in conjunction with periodontally accelerated osteogenic orthodontics. The treatment approach involved flap operation and selective partial decortication with alveolar bone grafting and augmentation. 3

Nevertheless, conventional corticotomy was considered invasive because the flap elevation often caused discomfort for patients. Various surgical approaches have been introduced as minimally invasive techniques. 4 For example, Piezocision refers to a method in which a cutting tip, used with substantial irrigation, is employed to create incisions in the cortical bone through the soft tissue. 5 Interseptal bone reduction involves preserving the cortical plate, with bone reduction occurring in the interseptal bone adjacent to the postextraction alveolar bone. 6 Corticision is a cortical bone incision procedure that is minimally invasive and does not require flap elevation. 7 Additionally, micro-osteoperforations (MOPs) are described as procedures in which small pinhole perforations are made in the bone surrounding the teeth intended for orthodontic movement. 8

The MOP technique has been suggested as a minimally invasive approach to accelerate orthodontic treatment in both animal and human studies. The induction of transient osteopenia through the creation of perforations in the cortical bone within the path of the targeted teeth reduces bone density, thereby facilitating more rapid tooth movement. 9 This procedure involves perforating the alveolar bone, which induces bone remodeling without flap operation. Transmucosal perforations of the cortical bone are created using the Propel system, Lance drill, or mini-implant. Performing MOPs in a human trial setting has shown that drilling into the bone using the Propel system at the extraction site effectively raised cytokine and chemokine expression. This biochemical reaction recruits and differentiates osteoclast precursors that increase the rate of tooth movement in canine retraction by 2.3 times versus controls. Additionally, patients who underwent MOPs experienced mild discomfort only at the perforation site, thus concluding that MOPs are efficient, convenient, and safe as a routine procedure. 8 10

The preferred depth of MOPs depends on the thickness of the gingiva and cortical plate. 11 When a premolar was extracted and canine retraction was subsequently performed to close the space, the typical surgical sites involved were the canine and the extraction site in the premolar area. Therefore, this systematic review and meta-analysis was divided into two depth ranges based on the thickness of the gingiva and cortical plate. A depth of 2 to 4 mm represents penetration through the cortical bone, which reaches the medullary bone, while a depth of 5 to 7 mm indicates penetration confined to the medullary bone only.

Several studies indicated that the MOPs can decrease treatment duration by accelerating tooth movement, but some complications were related to the periodontium, pain perception, quality of life, root resorption, and anchorage loss. 12 13 14 15 16 Some studies compared different devices for performing MOPs. Although subgroup analyses of MOPs with two and three holes were reported in a review, 15 no data were available regarding the effect of various depths of MOP perforation. Furthermore, canine retraction is a specific dental procedure that can be easily quantified. Additionally, numerous randomized controlled trials (RCTs) have been published on this issue. 12 The main purpose of this systematic review and meta-analysis was to provide a comprehensive analysis of the MOP field and to critically evaluate the current evidence supporting the intervention. The strength of this review lies in the inclusion of articles with similar characteristics, aiming to reduce clinical and statistical heterogeneity and enhance the reliability of the results. This systematic review and meta-analysis aimed to compare the clinical effectiveness of various depths of MOPs in accelerating the canine retraction rate and root resorption in orthodontic patients.

## Methods

The study was performed according to the guidelines of the Preferred Reporting Items for Systematic Reviews and Meta-Analyses (PRISMA) statement. 17 The prespecified protocol was registered in PROSPERO (CRD42024555722).

### Searches

Relevant literature was sought using a prespecified search strategy up to May 2024 ( Supplementary Appendix S1 , available in the online version only). Electronic medical and scientific databases included PubMed/MEDLINE, Scopus, EMBASE, Web of Science, and the Cochrane's Library (clinical trials).

- Condition or domain being studied: Patients who underwent fixed orthodontic treatment.
- Participants/population: Orthodontic patients of all ages who needed to undergo extraction of the maxillary first premolars followed by distalization of the maxillary canines.
- Intervention(s): MOPs.
- Comparator(s)/control: Conventional orthodontic treatment.

### Inclusion Criteria

A human study of any population size was included, and each study was assessed based on the following criteria:

1. RCTs
2. Studies that included patients of all ages who underwent orthodontic tooth movement acceleration with MOPs using any type of appliance
3. Studies that included patients who required premolar extractions and subsequent canine retraction

### Exclusion Criteria

1. Studies published in languages other than English
2. Studies involving both the intervention and control groups were included in the studies unless the outcomes for the intervention patients could be separated

### Main Outcome

Canine retraction rate (mm/month), root resorption (mm).

### Measures of Effect

Weighted mean difference (WMD).

### Data Extraction (Selection and Coding)

The search involved screening titles and abstracts of relevant literature found in databases that included PubMed/MEDLINE, Scopus, EMBASE, Web of Science, and the Cochrane's Library (clinical trials) up to May 2024. The inclusion of studies in the systematic review was determined by two reviewers using specific criteria. Two reviewers independently screened the records for inclusion. Any conflicts between individual decisions were resolved by a third reviewer. The means of recording data were recorded in an Excel spreadsheet.

For studies with incomplete outcome data, we contacted the corresponding author through e-mail. If no response was received within 2 weeks, a reminder was sent. If a response was not received after the second email, the data were noted as missing.

### Risk of Bias (Quality) Assessment

Two reviewers independently evaluated the risk-of-bias. Any conflicts in the quality assessment were discussed with the third reviewer. The Cochrane Collaboration's Risk-of-Bias 2 (RoB2) assessment tools were employed to evaluate the quality of all RCTs. The tools were assessed in five domains as follows: (1) bias from the randomization procedures, (2) deviations from intended interventions, (3) missing outcome data, (4) outcome measurement, and (5) selecting reported results. The studies were assessed and categorized into “low risk-of-bias,” “high risk-of-bias,” or “some concerns.”

### Strategy for Data Synthesis

A qualitative synthesis or systematic review that included the literature was reported before the quantitative synthesis. We assessed each study on both clinical and methodological heterogeneity to examine for transitivity and trial homogeneity. A pairwise meta-analysis was performed in the quantitative analysis to compare the effectiveness of treatments and evaluate any existing heterogeneity for treatment pairs with included studies more than one. Since the treatment outcome of interest was included, the canine retraction rates were treated as continuous data and the results were combined using WMD with 95% confidence intervals (CIs). The DerSimonian and Laird random-effects approach was applied to combine the effect estimates. 18 Statistical significance was determined by *p* -values of < 0.05. When a meta-analysis includes over 10 studies assessing the same interventions, publication bias would be assessed through funnel plots.

### Sensitivity and Subgroup Analysis

When heterogeneity was unacceptably high ( *I* ^2^ > 75%), the source was identified through subgroup analysis. Subgroup analysis was used to identify factors that modified the effects and influenced heterogeneity. A sensitivity analysis was performed to evaluate the robustness of the meta-analysis by removing high risk-of-bias studies, which could potentially bias the study results.

### Strength of Evidence

The Grading Quality of Evidence and Strength of Recommendations (GRADE) system was used to rate the strength of evidence of pairwise meta-analytic results by assessing the risk-of-bias in individual studies, the inconsistency, the indirectness, the imprecision, and any reporting biases. 19

## Results

**Fig. 1:**
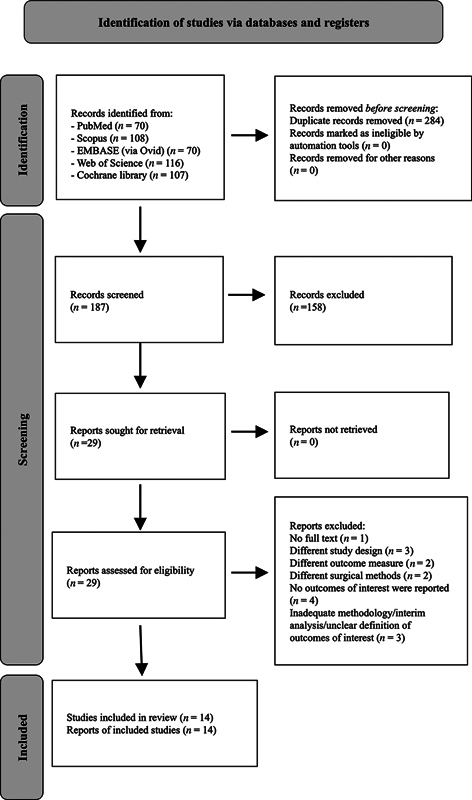
Flow diagram.

According to the search strategy, the search results from five databases consisted of 70 from PubMed, 108 from Scopus, 70 from EMBASE, 116 from Web of Science, and 107 from the Cochrane Library. Initially, 471 articles were discovered in the literature search, and 284 duplicated articles were excluded. However, after reviewing topics and abstracts, 158 articles were found unsuitable based on the research inclusion criteria. Following the reading of the 29 remaining full texts and the exclusion of those not meeting the inclusion criteria, 14 studies were deemed eligible for consideration ( Fig. 1 and Supplementary Appendix S2 [available in the online version only]).

### Characteristics of the Studies

The asymmetrical distribution in the funnel plots suggested publication bias. This could be attributed to trials with higher rates of canine retraction that had lower standard errors ( Supplementary Fig. S1 , available in the online version only).

The characteristics of the comparative articles that met the criteria are detailed in Table 1 . Fourteen RCT studies were included. The rate of canine movement was investigated in 14 studies, 10 20 21 22 23 24 25 26 27 28 29 30 31 32 while root resorption was investigated in 4 studies. 21 22 23 31 The selected articles were published between 2013 and 2022 with sample sizes that ranged from 8 to 32 individuals. The average age of the sample group ranged from 12.56 to 40 years old.

**Table 1:** Characteristics of the studies

Studies	Trial design	Age (y)	Sample size	Malocclusion	Surgical methods	Force activation	Anchorage	Measurement	Rate of canine retraction	Root resorption
Alikhani et al 10 (2013)	RCT	19.5–33.1	20	- Class II division 1 malocclusion	- Maxillary canine retraction - Using Propel	Nickel- titanium coil closing springs 100 g	Temporary anchorage device	Dental casts with an electric digital caliper	1 month MOPs: 1.27 ± 0.15 mm Control: 0.55 ± 0.15 mm	N/A
Haliloglu-Ozkan et al 26 (2018)	RCT	MOPs; 15.27 ± 1.62, Control; 16.13 ± 1.28	32 (19 M, 13 F)	N/R	- Maxillary and mandibular canine retraction - Using miniscrew - Distal of canine - 5 mm depth - Repeated in the fourth week of distalization	Nickel- titanium coil closing springs 150 g	Temporary anchorage device	Dental cast with digital caliper	1 month MOPs: 1.76 ± 0.66 mm Control: 1.36 ± 0.81 mm	N/A
Babanouri et al 24 (2020)	RCT (split- mouth)	16.3–35.2	25 (11 M, 14 F)	- Bimaxillary protrusion - Class II division 1 malocclusion	- Maxillary canine retraction - Using miniscrew - Middle of the extraction space - 1.2 mm width - 1 mm depth	Nickel- titanium coil closing springs 150 g	Temporary anchorage device	Digital models with digital caliper	1 month MOPs: 0.94 ± 0.31 mm Control: 0.64 ± 0.12 mm	N/A
Alkebsi et al 21 (2018)	RCT	19.26 ± 2.48	32 (8 M, 24 F)	- Class II division 1 malocclusion	- Maxillary canine retraction - Distal of canine - Using miniscrew - 5 mm depth - 1.5 mm width	Nickel- titanium coil closing springs 150 g	Temporary anchorage device	Digital models and digital caliper	1 month MOPs: 0.65 ± 0.26 mm Control: 0.67 ± 0.34 mm	3 month MOPs: –0.61 ± 2.11 mm Control: –0.73 ± 2.55 mm
Abdelhameed et al 20 (2018)	RCT (Three parallel groups)	15–25	30	- Dental full unit class II canine relationship - Bimaxillary protrusion	- Maxillary canine retraction - Using miniscrew - 1.6 mm width - 6 mm depth	Nickel- titanium coil closing springs 150 g	Temporary anchorage device	Direct intraoral measurement with digital caliper	1 month MOPs: 2.16 ± 0.27 mm Control: 1.31 ± 0.23 mm	N/A
Sivarajan et al 30 (2019)	RCT (split- mouth)	22.2 ± 3.72	30 (7 M, 23 F)	- Molar relationship < unit Class II or Class III	- Maxillary and mandibular canine retraction - Using miniscrew - Middle of extraction space - 3 mm depth - 1.6 mm width	Elastomeric chain 140–200 g	Temporary anchorage device	Direct clinical measurement with a digital caliper	−	N/A
Alqadasi et al 22 (2019)	RCT (split- mouth)	15–40	8 (4 M, 4 F)	- Class II division 1 malocclusion	- Maxillary canine retraction - Using miniscrew - Middle of the extraction space - 3 small perforations on buccal bone - 5–7 mm depth - 1.5–2 mm width	Nickel- titanium coil closing springs 150 g	Temporary anchorage device	Digital models	1 month MOPs: 1.11 ± 1.26 mm Control: 1.17 ± 0.72 mm	3 month MOPs: –0.03 ± 0.73 mm Control: –0.05 ± 1.1 mm
Alqadasi et al 23 (2021)	RCT (split- mouth)	20.89 ± 4.46	10 (4 M, 6 F)	- Class II division 1 malocclusion	- Maxillary canine retraction - Middle of the extraction space - Using miniscrew - 5–7 mm depth - 1.5–2 mm width	Nickel- titanium coil closing springs 150 g	Temporary anchorage device	Digital models	1 month MOPs: 1.07 ± 1.2 mm Control: 1.15 ± 0.7 mm	3 month MOPs: –0.04 ± 0.04 mm Control: –0.06 ± 0.09 mm
Thomas et al 31 (2021)	RCT (split- mouth)	19–25	30	- Class II division 1 malocclusion - Bimaxillary protrusion	- Maxillary canine retraction - Mesial and distal aspect of the canine root - Using a Lance drill - 2 mm width - 4 mm depth	Nickel- titanium coil closing springs 150 g	Temporary anchorage device	Direct clinical measurement with a digital caliper	1 month MOPs: 1.32 ± 0.4 mm Control: 0.86 ± 0.4 mm	3 month MOPs: –0.24 ± 1 mm Control: –0.3 ± 0.9 mm
Ozkan and Arici 28 (2021)	RCT	MOPs; 17.27 ± 1.22, Control; 18.13 ± 1.28	24 (12 M, 12 F)	- Class I malocclusion - Class II division 1 malocclusion	- Maxillary canine retraction - Using miniscrew - 4 and 7 mm depth - Diameter of 1.6 mm	Nickel- titanium coil closing springs 150 g	Temporary anchorage device	Digital models and digital caliper	1 month MOPs (4 mm): 1.22 ± 0.29 mm MOPs (7 mm): 1.3 ± 0.31 mm Control: 0.88 ± 0.2 mm	N/A
Golshah et al 25 (2021)	RCT (split- mouth)	16–25	25 (14 M, 11 F)	- Class II division 1 malocclusion	- Maxillary canine retraction - Using miniscrews with handpiece - Diameter of 1.6 mm - Depth in bone of 3–4 mm	Nickel- titanium coil closing springs 150 g	Temporary anchorage device	Digital models	1 month MOPs: 1.45 ± 0.65 mm Control: 1.23 ± 0.73 mm	N/A
Venkatachalapathy et al 32 (2022)	RCT (split- mouth)	15–25	20	- Class I molar and canine relationship - Bimaxillary protrusion	- Maxillary and mandibular canine retraction - Using miniscrews - Distal of canine - 3 mm in depth - 1.5 mm in width	Nickel- titanium coil closing springs 100 g	Temporary anchorage device	Dental cast with digital caliper	1 month MOPs: 0.65 ± 0.21 mm Control: 0.37 ± 0.09 mm	N/A
Raghav et al 29 (2022)	RCT (split- mouth)	20.32 ± 1.96	30	- Class II division 1 malocclusion - Bimaxillary protrusion	- Maxillary canine retraction - Using the Lance pilot drill - Distal to maxillary canine - Depth of 5 mm - Width of 2 mm	Nickel- titanium coil closing springs 150 g	Nance palatal button	Dental cast with digital caliper	1 month MOPs: 1.12 ± 0.49 mm Control: 0.82 ± 0.42 mm	N/A
Li et al 27 (2022)	RCT (split- mouth)	12.56–25.89	20 (9 M, 11 F)	N/R	- Maxillary canine retraction - Using Propel - Distal of canine - Depth of 5 mm	Nickel- titanium coil closing springs 150 g	Nance-transpalatal arch	Dental cast with digital caliper	1 month MOPs: 1.28 ± 0.56 mm Control: 1.16 ± 0.66 mm	N/A

Abbreviations: F, females; M, males; MOP, micro-osteoperforation; N/A, not assessed; N/R, not reported; RCT, randomized controlled trial.

From all of the included articles, reported depths of MOPs ranged from 1 to 7 mm. Ten articles 10 21 22 23 24 26 28 29 30 31 reported MOPs with three holes while one article 27 reported two holes and two articles 25 32 reported five holes. Additionally, one article reported 12 holes 20 ( Table 1 ).

### Effect of Interventions

#### Canine Retraction Rate at One Month

Thirteen articles 10 20 21 22 23 24 25 26 27 28 29 31 32 included in the analysis evaluated the impact of different depths of MOPs on the canine retraction rate over a period of 1 month. Only one study examined MOPs at a 1-mm depth within the cortical bone. The outcomes indicated that MOPs were effective in accelerating orthodontic tooth movement, but the increase was not clinically significant for the retraction of canines. 24

Another study involved three separate groups to investigate the effectiveness of MOPs at a depth of 3 mm. Their research focused on measuring the extent of canine retraction over a period of 16 weeks. The results indicated that all MOP groups demonstrated significantly greater canine distalization compared with the control groups. 30 An additional article that used MOPs at a depth of 2 to 3 mm resulted in a 2.3-fold increase in the canine retraction rate, which was significantly higher than the control group and the opposite side of the experimental group. 10

**Fig. 2:**
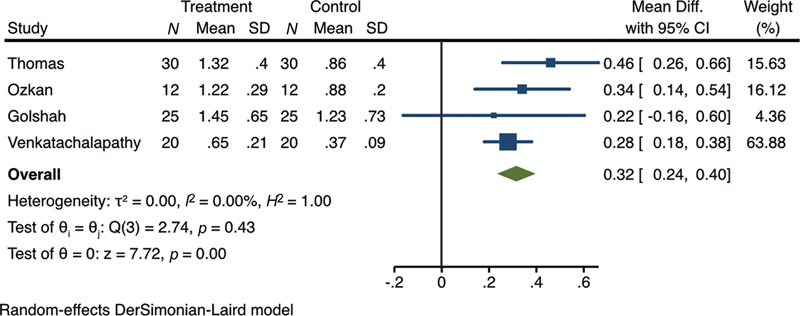
Forest plot of the comparison of canine retraction rate at 1 month between micro-osteoperforations (MOPs) at depths of 2 to 4 mm and the controls.

We grouped the various depths of MOPs into two groups: 2 to 4 and 5 to 7 mm. The meta-analysis on MOPs at depths of 2 to 4 mm and their impact on the canine retraction rate showed that the MOP groups had a significantly higher rate compared with the control groups (WMD = 0.32; 95% CI, 0.24–0.40; *p* = 0.00) ( Fig. 2 ).

**Fig. 3:**
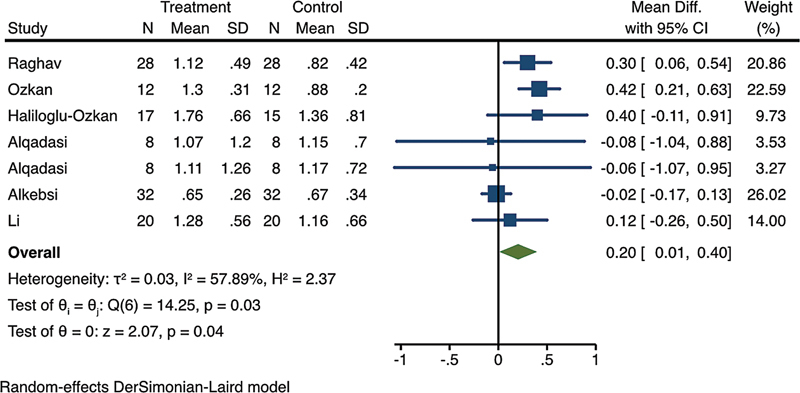
Forest plot of the comparison of canine retraction rate at 1 month between micro-osteoperforations (MOPs) at depths of 5 to 7 mm and the controls.

Moreover, the meta-analysis results on MOPs at depths of 5 to 7 mm revealed that the MOP groups had a significantly higher rate compared with the control groups (WMD = 0.20; 95% CI, 0.01–0.40; *p* = 0.04) ( Fig. 3 ).

##### Sensitivity Analysis

A sensitivity analysis examined the potential factors that could bias the study results by removing the high risk-of-bias studies. The meta-analysis results on MOPs at depths of 2 to 4 and 5 to 7 mm revealed that the experimental groups still exhibited significantly higher rates compared with the control groups. The sensitivity analysis demonstrated that the results were robust at both depths ( Supplementary Figs. S2 and S3 , available in the online version only).

#### Root Resorption

The assessment of root resorption was reported in four articles. Because of methodological inconsistencies and incoherence of the assessed studies, it was not possible to conduct a quantitative assessment of the extracted data. Three studies that utilized cone-beam computed tomography reported no differences between both sides (MOPs and control). 22 23 31 More root resorption occurred on the control side than on the MOP side after 3 months. Only one study investigated root resorption using periapical radiographs. The results showed substantial root resorption in both the MOP and control groups after 3 months. However, a statistically significant difference was not found between the control and MOP sides. 21

### Risk-of-Bias Assessment

According to the Cochrane RoB2 assessments, nine studies were identified as having a high risk-of-bias, while the other five studies were reported with some concerns ( Supplementary Fig. S4 , available in the online version only). The studies were considered at high risk-of-bias due to concerns about potential bias in the outcome measurement. Blinding participants and operators was not feasible due to the nature of surgical procedures. Nonetheless, studies were rated as low risk-of-bias if blinding was implemented during data collection. The proportions summarized for each domain of RoB2 are illustrated in Supplementary Fig. S4 , available in the online version only.

### Strength of Evidence

The strength of evidence from the pairwise meta-analysis was summarized separately for canine retraction rates at depths of 2 to 4 and 5 to 7 mm with both rated as low quality. The GRADE profiles for these rates were downgraded due to the risk-of-bias and inconsistency. The domain of risk-of-bias was downgraded because several RCTs had a high risk-of-bias. Additionally, the inconsistency domain was downgraded due to high heterogeneity in both pairwise meta-analyses ( Table 2 ).

**Table 2:** GRADE evidence profile of the canine retraction rates

Certainty assessment							No. of patients		Effect		Certainty	Importance
No. of studies	Study design	Risk of bias	Inconsistency	Indirectness	Imprecision	Other considerations	MOPs	Control	Relative (95% CI)	Absolute (95% CI)		
Canine retraction rate at depths of 2–4 mm												
5	RCTs	Serious a	Serious b	Not serious	Not serious	None	107	107	−	MD 0.32 higher(0.24 higher to 0.4 higher)	⨁⨁◯◯ Low	Critical
Canine retraction rate at depths of 5–7 mm												
8	RCTs	Serious a	Serious b	Not serious	Not serious	None	134	132	−	MD 0.2 higher (0.01 higher to 0.4 higher)	⨁⨁◯◯ Low	Critical

Abbreviations: CI, confidence interval; GRADE, Grading Quality of Evidence and Strength of Recommendations; MD, mean difference; MOP, micro-osteoperforation; RCT, randomized controlled trial.

a Most studies were at high risk-of-bias.

b Inconsistency in results across the included studies.

## Discussion

All studies included participants across a wide age range. Typically, participant ages ranged from 12.56 years as the minimum to 40 years as the maximum, which suggested that these findings may be applied to adolescents and adults. Age is an important factor in orthodontic tooth movement. Adults exhibited slower tooth movement compared with adolescents, especially during canine distalization. 33 However, most of the included studies are split-mouth, randomized controlled clinical trials, which help minimize age-related bias that could influence the study outcomes. Moreover, there were various measurements for canine movement, but we judged that the different methods of measurement were suitable for pooling the results in the same unit of measurement.

Although previous studies have indicated that surgical adjunctive procedures can accelerate orthodontic tooth movement and shorten treatment duration, the acceleration is minor, temporary, and based on low-level evidence. Therefore, a cost–benefit analysis of these procedures should be taken into account when making treatment decisions. 34 However, this study demonstrated the significant effectiveness of MOPs in accelerating orthodontic treatment compared with conventional methods, in particular regarding the canine retraction rate. The depth of MOPs at 2 to 4 mm was optimal for accelerating orthodontic treatment. Similarly, depths between 5 and 7 mm also provided favorable outcomes. The results of the pairwise meta-analysis that were obtained were consistent with previous studies, which indicated a significantly higher canine retraction rate per month in the MOP groups. 15

However, when comparing the effectiveness of perforations at 2 to 4 mm with 5 to 7 mm, it was found that perforations at 2 to 4 mm were more effective. Hence, it can be concluded that more invasive perforations did not effectively promote tooth movement. Similar to a recent study that investigated the effect of different MOP depths on the canine retraction rate at depths of 4 and 7 mm, the study found that the two depths were not significantly different. Additionally, both groups showed significantly increased canine movement compared with a control group. 28

After the MOP procedure, bone injury stimulates the release of cytokines, which accelerate bone turnover that leads to a reduction in regional bone density. 2 This phenomenon is known as the RAP that usually initiates shortly after the surgical injury and peaks within 1 to 2 months of the surgical intervention. 3 A comparison with other surgically assisted orthodontic methods in this study showed that MOPs led to less canine retraction compared with corticotomy and Piezocision but was higher than vibration and low-level laser therapy during the first month. 35 This situation suggests that the extent of the surgery correlates with RAP. However, MOPs still yield accelerating movement of the canine and can be applied in routine practice. The MOP procedure follows the Biphasic Theory of Tooth Movement, which involves two consecutive alveolar bone remodeling phases triggered by orthodontic force: the catabolic phase followed by the anabolic phase. The minor trauma to the bone caused by MOPs releases inflammatory markers that induce a catabolic effect that activates osteoclasts and promotes bone resorption. Nevertheless, osteoblasts replace osteoclasts, which mark the onset of a repair phase that reconstructs the resorbed bone structure. This stage is recognized as the anabolic phase. 36 37 38

Heterogeneity arises from clinical heterogeneity, and studies should be selected based on similar populations that can be observed from demographic data. Methodological heterogeneity can also emerge even when the interventions being studied are similar. This variation may be due to differences in measurement methods, the personnel conducting the measurements, and the specific techniques employed for data collection. Methodological heterogeneity was apparent in the MOP studies with variations in sample sizes, ages of the samples, severity of malocclusion, surgical protocols, force application methods, and the types of anchorage affecting the rates of canine retraction. Most included RCT studies could not blind the patient or the clinician who performed the MOP procedure because of the nature of the study. In this study, we chose to examine the canine retraction rate at 1 month to reduce bias from repeated interventions observed in some studies. The sensitivity analysis, which removed the high risk-of-bias studies, indicated a decrease in heterogeneity and confirmed the robustness of the study findings in the two depth groups.

In three studies, no differences were observed between both sides, that is, the MOP side and the control side. However, increased root resorption was noted on the control side compared with the MOP side 3 months postsurgery, 22 23 31 possibly attributable to corticotomy procedures that reduced bone density and thereby accelerated tooth movement. 39 Orthodontic tooth movement requires the lamina dura beside the periodontal ligament (PDL) to undergo osteoclast formation on the pressure side of the root. When hyalinization necrosis occurs, osteoclastic activity in the affected PDL region ceases. After 3 to 5 weeks, the damaged tissue will be removed. There is a reduction in cellular function and blood flow in both the PDL and adjacent bone. Osteopenia caused by RAP enhances osteoclastic activity and reduces bone density, thereby lowering the chances of hyalinization necrosis and root resorption. 40

The GRADE approach to rating the quality of evidence and making recommendations resulted in a low quality assessment in both outcomes. Despite a downgrade in three out of five domains, it maintains a transparent framework for grading evidence and interventions that demands considerable resources. Pooling resource estimates from diverse studies are rarely undertaken due to potential controversies involved and the necessity for careful consideration.

Based on the results of the funnel plot, an asymmetrical distribution was observed, which suggested the presence of potential publication bias. A funnel plot was performed to determine if publication bias affected the observed effect and to estimate the effect size without bias. The distribution of the mean possible from this population was precise. The low level of variability in the data supports the conclusion that the selected articles were of relatively high quality. However, the included studies in this meta-analysis with smaller sample sizes have a greater distribution of the mean.

The study demonstrated the clinical relevance of MOP intervention depths, showing that both the 2 to 4 and 5 to 7 mm depths accelerated canine retraction compared with the controls, but only by 0.32 and 0.20 mm/month, respectively. This finding facilitates clinicians in evaluating the risk–benefit of utilizing surgical intervention during orthodontic tooth movement. However, both MOP intervention depths exhibited root resorption, which was not significantly different from that observed in the controls.

### Limitations

Non-English articles were excluded from this review, which possibly led to missing data. A variety of surgical protocols, such as appliance size and type, can lead to heterogeneous results. Few studies were available, and the range of MOP depths largely varied. The diversity of surgical protocols also made the data analysis more challenging. Additionally, a variety of reference points and measurement techniques were used in each study.

### Further Study

- More clinical trials are required to compare MOPs due to limited evidence on the impacted factors on accelerated tooth movement.
- More research is required to investigate the canine retraction rate after the first month and potentially assess the overall rate thereafter.
- The narrower age range distribution of the population specified in the Population, Intervention, Comparison, and Outcome framework should be explored in further studies.
- Other methods of orthodontic acceleration, such as photobiomodulation, should be included in future investigations.

## Conclusion

- Both depths of MOPs, that is, 2 to 4 and 5 to 7 mm, promoted acceleration of canine retraction more than the controls by approximately 0.32 and 0.20 mm/month, respectively.
- However, both depths of MOPs presented root resorption during canine retraction that were not different from the controls.
