# Supplementary material for: Effects of Micro-Osteoperforation Depths on Canine Retraction Rate and Root Resorption: A Systematic Review and Meta-Analysis
Source: Eur J Dent. 2025 May 2;19(4):893–902. doi: 10.1055/s-0045-1806932 (PMC12494454; doi:10.1055/s-0045-1806932)
Supplement: Supplementary file 1 — Supplementary Material [file 10-1055-s-0045-1806932-s24113899.pdf]

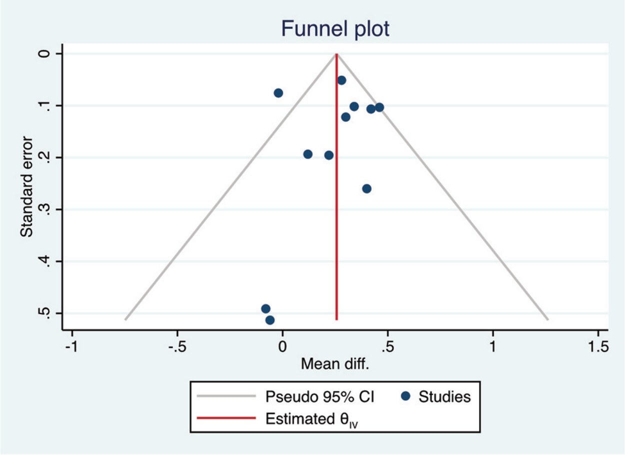

Supplementary Fig. S1 Funnel plot assessing the overall publication bias of the included studies.

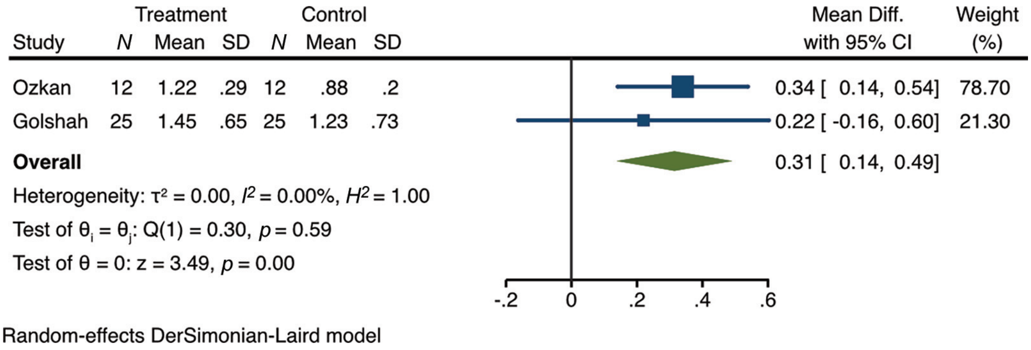

Supplementary Fig. S2 Forest plot of the sensitivity analysis of canine retraction rate at 1 month between micro-osteoperforations (MOPs) at depths of 2 to 4 mm and the controls.

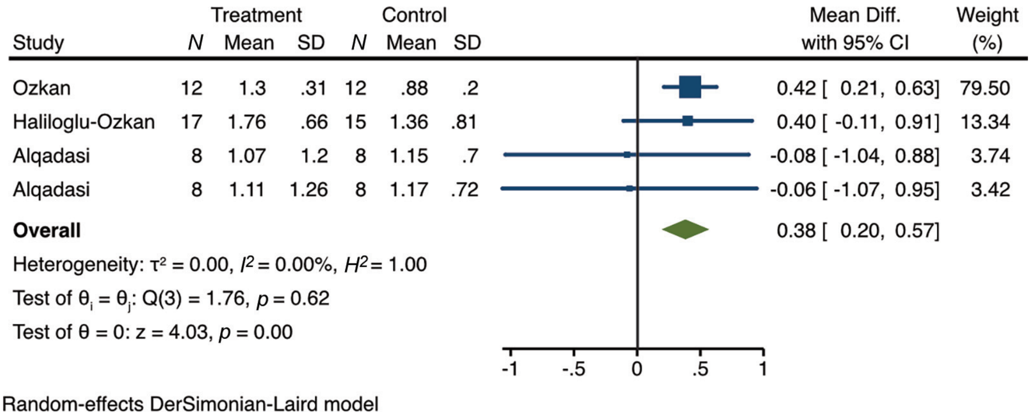

Supplementary Fig. S3 Forest plot of sensitivity analysis of canine retraction rate at 1 month between micro-osteoperforations (MOPs) at depths of 5 to 7 mm and the controls.

 © 2025. The Author(s).

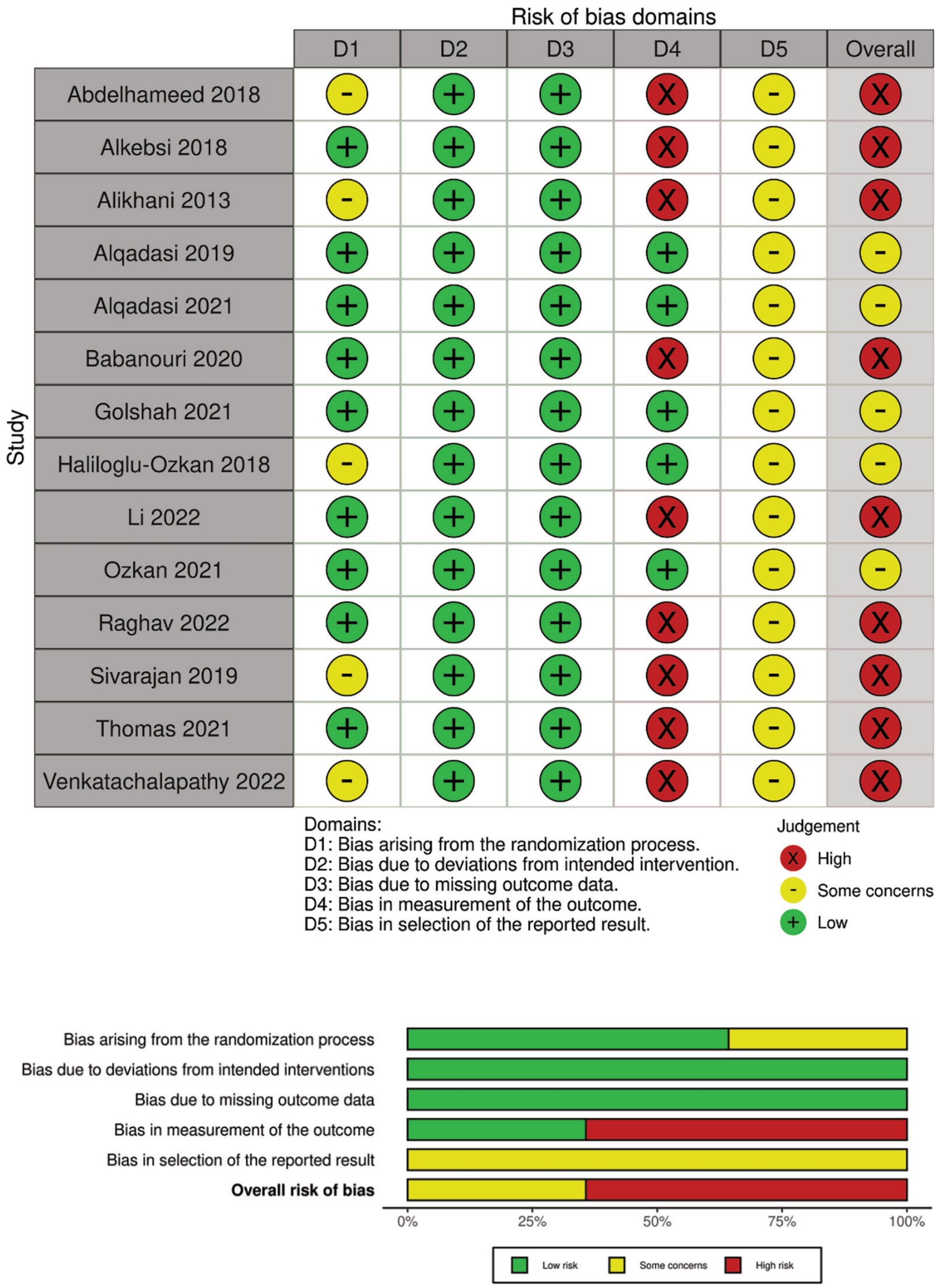

Supplementary Fig. S4 Risk-of-bias assessment of the included studies.

 © 2025. The Author(s).

Supplementary Appendix S1 Search strategy

Databases	Search strategy	Result
PubMed	#1 “Orthodontic*”[tw]<br>#2 “Malocclusion”[tw]<br>#3 “Micro-osteoperforation*”[tw]<br>#4 “Osteoperforation*”[tw]<br>#5 “Osteopuncture “[tw]<br>#6 “Retraction rate”[tw]<br>#7 “Tooth movement “[tw]<br>#8 “Canine movement”[tw]<br>#9 #1 or #2<br>#10 #3 or #4 or #5<br>#11 #6 or #7 or #8<br>#12 #9 and #10 and #11	65969<br>37499<br>82<br>96<br>3<br>111<br>11990<br>70<br>82754<br>99<br>12086<br>70
EMBASE via Ovid	1 Orthodontic*<br>2 Malocclusion<br>3 Micro-osteoperforation*<br>4 Osteoperforation*<br>5 Osteopuncture<br>6 Retraction rate<br>7 Canine movement<br>8 Tooth movement<br>9 1 or 2<br>10 3 or 4 or 5<br>11 6 or 7 or 8<br>12 9 and 10 and 11	48504<br>23726<br>75<br>92<br>2<br>110<br>58<br>5249<br>58392<br>94<br>5355<br>70
Scopus	((TITLE-ABS-KEY (“Orthodontic*”)) OR (TITLE-ABS-KEY (“ Malocclusion”))) AND ((TITLE-ABS-KEY (“Micro-osteoperforation*”)) OR (TITLE-ABS-KEY (“Osteoperforation*”)) OR (TITLE-ABS-KEY (“Osteopuncture”))) AND ((TITLE-ABS-KEY (“Retraction rate”)) OR (TITLE-ABS-KEY (“Canine movement”)) OR (TITLE-ABS-KEY (“Tooth movement”)))	108
The Cochrane Library	#1 Orthodontic*<br>#2 Malocclusion<br>#3 Micro-osteoperforation*<br>#4 Osteoperforation*<br>#5 Osteopuncture<br>#6 Retraction rate<br>#7 Canine movement<br>#8 Tooth movement<br>#9 #1 or #2<br>#10 #3 or #4 or #5<br>#11 #6 or #7 or #8<br>#12 #9 and #10 and #11	6816<br>2238<br>126<br>130<br>2<br>1098<br>463<br>1591<br>7436<br>132<br>2499<br>107
Web of Science	1 “Orthodontic*”<br>2 “Malocclusion”<br>3 “Micro-osteoperforation*”<br>4 “Osteoperforation*”<br>5 “Osteopuncture “<br>6 “Retraction rate”<br>7 “Tooth movement “<br>8 “Canine movement”<br>9 #1 or #2<br>10 #3 or #4 or #5<br>11 #6 or #7 or #8<br>12 #9 and #10 and #11 Timespan: until 2024–05–31	35481<br>11573<br>137<br>154<br>1<br>166<br>68<br>5744<br>41016<br>155<br>5905<br>116

Supplementary Appendix S2 List of excluded studies with reason

Reference	Reason for exclusion
Aboalnaga[1]	Inadequate methodology/unclear definition of outcomes of interest
Agrawal[2]	Different study design
Attri[3]	Different outcome measure
Bajaj[4]	Different surgical methods
Bolat Gümüş[5]	No outcomes of interest were reported
Farag[6]	Different outcome measure
Fattori[7]	Different study design
Jaiswal[8]	No outcomes of interest were reported
Kundi[9]	No full text
Prasad[10]	No outcomes of interest were reported
Teh[11]	Different surgical methods
Hashem[12]	No outcomes of interest were reported
Martina[13]	Unclear definition of outcomes of interest
Mehta[14]	Inadequate methodology/unclear definition of outcomes of interest
Shahrin[15]	Different study design

 © 2025. The Author(s).

1 Aboalnaga AA, Salah Fayed MM, El-Ashmawi NA, Soliman SA. Effect of micro-osteoperforation on the rate of canine retraction: a split-mouth randomized controlled trial. Prog Orthod 2019;20(01):21
2 Agrawal AA, Kolte AP, Kolte RA, Vaswani V, Shenoy U, Rathi P. Comparative CBCT analysis of the changes in buccal bone morphology after corticotomy and micro-osteoperforations assisted orthodontic treatment - case series with a split mouth design. Saudi Dent J 2019;31(01):58–65
3 Attri S, Mittal R, Batra P, et al. Comparison of rate of tooth movement and pain perception during accelerated tooth movement associated with conventional fixed appliances with micro-osteoperforations - a randomised controlled trial. ArticleJ Orthod 2018;45(04):225–233
4 Bajaj I, Garg AK, Gupta DK, Singla L. Comparative effect of micro-osteoperforation and Photo-biomodulation on the rate of maxillary canine retraction: a split mouth randomized clinical trial. Clin Ter 2022;173(01):39–45
5 Bolat Gümüş E, Kınsız E. Effects of miniscrew-facilitated micro-osteoperforations on the rate of orthodontic tooth movement : a split-mouth, randomized controlled trial. J Orofac Orthop 2023;84(Suppl 2):104–110
6 Farag T, Refai WMM, Nasef A, Elhiny OA, Hashem AS. Evaluation of the effect of micro-osteoperforations versus piezopuncture on the rate of orthodontic tooth movement associated with canine retraction. ArticleOpen Access Maced J Med Sci 2021;9(D):113–119
7 Fattori L, Sendyk M, de Paiva JB, Normando D, Neto JR. Micro-osteoperforation effectiveness on tooth movement rate and impact on oral health related quality of life. ArticleAngle Orthod 2020;90(05):640–647
8 Jaiswal AA, Siddiqui HP, Samrit VD, Duggal R, Kharbanda OP, Rajeswari MR. Comparison of the efficacy of two-time versus one-time micro-osteoperforation on maxillary canine retraction in orthodontic patients: a split-mouth randomized controlled clinical trial. Int Orthod 2021;19(03):415–424
9 Kundi I. Effect of flapless cortical perforation on canine retraction rate: a randomized clinical trial. ArticleInt Med J 2018;25(02):116–118
10 Prasad AS, Subramanian AK, Varghese RM. Comparison of mesial molar migration associated with different depths of micro-osteoperforation assisted canine retraction. Eur J Mol Clin Med 2020;7(02):242–250
11 Teh NHK, Sivarajan S, Asif MK, Ibrahim N, Wey MC. Distribution of mandibular trabeculae bone volume fraction in relation to different MOP intervals for accelerating orthodontic tooth movement. Angle Orthod 2020;90(06):774–782
12 Hashem BA, El-Hassanein EH, El-Awady AA, et al. Clinical Evaluation of Single Versus Repeated Micro-Osteoperforations During Orthodontic Canine Retraction: A Randomized Clinical Trial. Cureus 2024;16(01)
13 Martina K, Kumar PS, Misra V, et al. To evaluate the rate of canine retraction and pain perception following micro-osteoperforation - a split-mouth clinical study. Australas Orthod J 2022;38(02):388–395
14 Mehta A, Shah A, Patel V, Desai B, Patel R, Patel V. Effect of micro-osteoperforations on the rate of orthodontic tooth movement: a randomized controlled trial. J Contemporary Orthodont 2020;4(01):12–20
15 Shahrin AA, Ghani SHA, Norman NH. Effectiveness of micro-osteoperforations in accelerating alignment of maxillary anterior crowding in adults: a randomized controlled clinical trial. Am J Orthod Dentofacial Orthop 2021;160(06):784–792

 © 2025. The Author(s).
